# Immature defences mediate the association between borderline personality and problematic social media use in generation Z

**DOI:** 10.3389/frcha.2026.1822113

**Published:** 2026-06-23

**Authors:** Danilo Bontempo, Marco Giancola, Matteo Perazzini, Simonetta D'Amico, Enrico Perilli

**Affiliations:** 1 Department of Life, Health and Environmental Sciences, University of L'Aquila, L’Aquila, Italy; 2 Department of Biotechnological and Applied Clinical Sciences, University of L'Aquila, L’Aquila, Italy

**Keywords:** addiction, human-computer interaction, immature defence, mediation, mental health

## Abstract

**Introduction:**

Problematic Social Media Use (PSMU) is increasingly recognised as a relevant public mental health concern. Nevertheless, the main psychological mechanisms underpinning this pattern remain underexplored, especially in Generation Z, for whom social media represents a primary environment for identity construction, interpersonal interaction, information seeking, and self-expression. Grounded in the Interaction of Person–Affect–Cognition–Execution (I-PACE) framework and psychodynamic theory of defence mechanisms, the present study tested whether immature defences mediate the association between Borderline Personality and PSMU.

**Methods:**

Five hundred and twenty participants aged 18–25 years (M_age_ = 20.82, SD_age_ = 1.66; 252 females) who reported daily social media use completed an online survey, including a brief socio-demographic questionnaire, the Borderline Symptom List-23, the Defence Style Questionnaire-40, and the Bergen Social Media Addiction Scale.

**Results:**

Results indicated that immature defences mediated the association between Borderline Personality and PSMU, after controlling for age, sex, education, mature defences, and neurotic defences.

**Discussion:**

Overall, these findings suggest that immature defences may represent one psychological process linking Borderline Personality to PSMU, reflecting maladaptive, unconscious strategies of affect regulation. Limitations, theoretical avenues, and clinical implications are discussed.

## 1 Introduction

Social media has become an integral component of contemporary daily life, profoundly reshaping how individuals communicate, construct identity, and engage with social reality. Social media use spans across generational cohorts, including Baby Boomers (birth year range: 1946–1964), Generation X (birth year range: 1965–1979), Generation Y or Millennials (birth year range: 1980–1994), and Generation Alpha (birth year range: 2010–2025). However, converging evidence indicates that Generation Z (birth year range: 1995–2010) exhibits the highest levels of engagement with social media platforms (1). Unlike previous generations, Generation Z represents the first cohort of true digital natives, having been born into an environment characterised by ubiquitous internet access, mobile technologies, and constant connectivity (2). Exposure to this environment from early developmental stages has fostered advanced digital fluency with online interaction norms (1). Consequently, for Generation Z more than any other generation, social media platforms function as a central digital ecosystem for identity formation, interpersonal relationships, information seeking, and self-expression.

Despite their communicative and social benefits, increasing concern has emerged regarding compulsive, dysregulated, and maladaptive patterns of social media engagement, particularly among younger populations (3). Within this framework, Problematic Social Media Use (PSMU) refers to a maladaptive and dysregulated pattern of social media engagement that results in significant impairment in well-being, social functioning, and daily life activities (4). Conceptually grounded in addiction-related models (5, 6), PSMU is characterised by six core features: 1) salience (preoccupation with social media at the expense of other activities); 2) mood modification (using social media to regulate mood); 3) tolerance (needing increasing use of social media to achieve the same initial effect); 4) withdrawal (negative affective or somatic states when access to social media is restricted); 5) conflict (intrapersonal and interpersonal problems arising from excessive social media use), and 6) relapse (a return to problematic social media use after abstinence).

Given the adverse consequences associated with PSMU, a growing body of research has sought to identify the psychological mechanisms underlying individual vulnerability to this condition. Within this context, the present study investigates the association between Borderline Personality and PSMU, with a focus on the potential mediating role of immature defence mechanisms.

### 1.1 The association between borderline personality and PSMU

Within the literature on individual differences in PSMU, the Interaction of Person–Affect–Cognition–Execution (I-PACE) model (7–9) has been widely used as a scientific framework to generate research hypotheses and to explain empirical findings. In particular, the I-PACE model conceptualises PSMU as the result of dynamic interactions among person's core characteristics (P), affective and cognitive responses to specific stimuli or situations (A and C), and executive functions (E). Within the *P* component, personality has been identified as a key determinant in both the development and maintenance of PSMU.

Specifically, research grounded in both Big Five and HEXACO personality models has consistently reported negative associations between PSMU and Conscientiousness and Agreeableness, alongside positive relationships with Neuroticism and Openness to Experience (10–12). Meta-analytic evidence further indicated that Neuroticism and Conscientiousness together account for up to 91% of the variance in PSMU explained by the Big Five (13).

Beyond the Big Five and HEXACO models, increasing attention has been devoted to the Dark Side of Personality, namely maladaptive personality traits (14, 15). Research on Dark Triad/Dark Tetrad suggested that high levels in Machiavellianism, Narcissism, Psychopathy, and Sadism are associated with PSMU due to heightened impulsivity, emotion dysregulation, and a tendency toward exploitative social behaviours (16, 17). Extending this line of inquiry, emerging evidence on the Vulnerable Dark Triad (i.e., Vulnerable Narcissism, Secondary Psychopathy, and Borderline Personality) indicated that Borderline Personality can constitute one of the main salient risk factors for PSMU (18).

Borderline Personality is characterised by pervasive emotional instability, marked affective fluctuations, and recurrent episodes of intense sadness, anger, or anxiety (19). From an interpersonal standpoint, this trait involves unstable interpersonal relationships, intense attachments, and a pronounced fear of abandonment, often resulting in alternating patterns of idealisation and devaluation toward others (20). These vulnerabilities can predispose to addictive behaviours (e.g., alcohol dependence, gambling disorder, internet gaming disorder) as compensatory strategies to regulate affect, soothe relational insecurities, and maintain perceived social closeness (21–23).

Within the context of PSMU, core features associated with Borderline Personality, such as a persistent need for reassurance and validation, fear of exclusion, and heightened emotional lability, may predispose to compulsive checking of notifications, hypervigilant monitoring of online interactions, and excessive engagement with social media as a means of obtaining affirmation and attention from others (18, 24). According to this perspective, Borderline Personality can be viewed as a critical *P* factor within the I-PACE framework, contributing to the development and maintenance of PSMU through maladaptive emotion regulation processes and unstable interpersonal motivations.

### 1.2 The mediating role of the immature defence mechanisms

Despite the emerging evidence linking Borderline Personality and PSMU, the psychological mechanisms that may account for the association between this personality trait and compulsive and dysregulated social media use remain overlooked. In this regard, defence mechanisms (or defence styles) may represent a critical construct for elucidating this association.

The concept of defence mechanisms was first introduced by Freud (1894/1962) and constitutes a fundamental component of personality functioning. It refers to unconscious and automatic psychological mechanisms through which individuals manage internal conflicts, regulate painful affect, and cope with external stressful situations, thereby maintaining psychological stability (25). These mechanisms are hierarchically organised according to their level of maturity and adaptiveness in three broad styles: mature, neurotic, and immature defences (26, 27). According to Andrews et al. (28), mature defences (i.e., sublimation, humour, anticipation, and suppression) are the most adaptive and promote enjoyment while allowing for more cognitive and cohesive understanding of mental states and interpersonal experiences. Neurotic defences (i.e., undoing, pseudo-altruism, idealisation, and reaction formation) operate by partially excluding emotional or cognitive material from conscious awareness, allowing temporary containment of distress while preserving reality testing. In contrast, immature defences (i.e., projection, passive aggression, acting out, isolation, devaluation, autistic fantasy, denial, displacement, dissociation, splitting, rationalisation, and somatisation) are characterised by marked distortion of inner reality (e.g., unpleasant feelings) and external reality, limited symbolic elaboration of affect, and a predominance of action-oriented or perceptual strategies over reflective processing.

Within the relationship between Borderline Personality and PSMU, immature defences may be particularly relevant. Indeed, Borderline Personality is strongly associated with immature defences, which reflect low reality integration, externalisation of intrapsychic conflict, identity diffusion, and impaired affect modulation (29). Although immature defences may provide short-term relief from overwhelming emotional states and psychological distress, they can lead to long-term costs in terms of psychological instability, interpersonal functioning, and fragmented self-representation (30–32).

Importantly, these same defences are significant for the structural features of digital environments. Social media platforms are characterised by immediacy, ambiguity, constant social comparison, intermittent reinforcement, and emotionally salient feedback (33, 34). While the direct association between immature defence styles and PSMU has not yet been systematically investigated, these defence styles may be activated and reinforced within social media environments. For instance, denial and dissociation may facilitate prolonged immersion and disengagement from offline distress, while projection and devaluation may intensify hostile or conflictual online interactions. Idealisation may foster excessive attachment to online figures or feedback, acting out may manifest as impulsive posting or compulsive checking, while splitting may amplify polarised interpretations of online experiences. Consistent with this view, previous research has shown that immature defence styles are positively associated with various forms of problematic use of technologies, including problematic internet use (35).

From the I-PACE perspective, immature defences may be conceptualised as person-related regulatory mechanisms that influence affective and cognitive responses to emotionally salient situations. In individuals with high levels of Borderline Personality, distress arising from rejection sensitivity, perceived exclusion, relational uncertainty, or identity-related insecurity may activate immature defensive processes. These processes may, subsequently, facilitate the transition from internal emotional tension to external behavioural engagement with social media. In this direction, immature defences may be interpreted as regulatory processes operating between the *P* component and the affective-cognitive components of the I-PACE model, shaping how Borderline Personality vulnerabilities are associated with PSMU. Accordingly, immature defences may represent a psychological bridge through which Borderline Personality features are translated into PSMU.

Therefore, the research hypothesis was as follows: immature defences mediate the relationship between Borderline Personality and PSMU.

### 1.3 The present study

Drawing on the I-PACE model and research on individual differences in PSMU, the present study aimed to advance the understanding of the psychological mechanisms underlying PSMU by integrating personality traits and defence mechanisms. In particular, this research examined the association between Borderline Personality and PSMU and investigated whether immature defences mediate this relationship. By adopting this mediation approach, the study sought to move beyond the direct association between Borderline Personality and PSMU and to clarify how this personality trait may increase vulnerability to PSMU through maladaptive and unconscious affect-regulation strategies.

## 2 Method

### 2.1 Participants

The required sample size was determined based on the average effect size in personality psychology [r ≈ 0.20 (36);] and recommendations (*N* ≈ 250) for reducing estimation error in this research field (37). Five hundred and twenty-nine participants took part in the study. After removing participants with missing data (*N* = 3) and univariate outliers (*N* = 6) using ± 4 z-score as the threshold for samples larger than 100 participants (38–40), the final sample consisted of 520 participants aged between 18 and 25 years (M_age_ = 20.82, SD_age_ = 1.66; 252 females), who reported to use social media daily. All participants reported daily use of at least one social media platform and were Italian, reflecting a Western cultural background (see Table 1 for socio-demographic features of the sample). Participants were included in the study if they met the following inclusion criteria: 1) age between 18 and 25 years (consistent with Generation Z), 2) daily use of social media; 3) fluency in Italian; 4) absence of severe or currently diagnosed psychiatric and/or neurological conditions (e.g., psychotic disorders, traumatic brain injury, etc) that could interfere with the research.

**Table 1:** Socio-demographic features of the sample.

Variable	Frequency (%)	M (SD)	Min-Max
Age		20.82 (1.66)	18–25
Sex			
Male	268 (51.50)		
Female	252 (48.50)		
Education (years)		13.18 (0.97)	8–18
Occupation			
Student	380 (73.10)		
Working student	72 (13.80)		
Employed	50 (9.60)		
Unemployed	12 (2.30)		
Not declared	6 (1.20)		
Income (in Euros)			
< 10.000	82 (15.80)		
11.000–25.000	252 (48.50)		
26.000–50.000	122 (23.50)		
51.000–75.000	38 (7.30)		
76.000–100.000	12 (2.30)		
> 100.000	4 (0.80)		
Not declared	10 (1.90)		

*N* = 520.

### 2.2 Procedure

Data were obtained using an online cross-sectional survey administered via Google Forms from May 2024 to November 2024. Recruitment was conducted using a non-probabilistic snowball sampling method through online announcements disseminated across various social media platforms (Facebook, Instagram, and WhatsApp) and via interpersonal sharing. Before participation, all subjects were presented with a digital informed consent form describing the main aims and procedures of the study. Consent was provided electronically by selecting an agreement checkbox. Participation was voluntary and without any rewards. All data were collected anonymously, and participants were informed that their information would be treated confidentially and securely stored. The study protocol received approval from the Institutional Review Board (IRB) of the University of L'Aquila (Protocol No. 119943).

### 2.3 Measures

#### 2.3.1 Socio-demographic information

A brief socio-demographic questionnaire assessed age, biological sex, and educational level of the sample.

#### 2.3.2 Borderline personality

Borderline Personality was evaluated using the Borderline Symptom List [BSL-23 (41);]. The BSL-23 evaluates the severity of borderline symptomatology via 23 items (e.g., ‘I was absent-minded and unable to remember what I was actually doing’) on a 5-point Likert-type scale ranging from 0 (not at all) to 4 (very strong). As no officially validated Italian version was available at the time of data collection, an Italian adaptation was developed using a standard forward–backward translation procedure to ensure linguistic and conceptual equivalence with the original instrument. The forward translation was conducted from English into Italian, followed by an independent back-translation into English. Discrepancies between the original and back-translated versions were discussed and resolved by consensus. In this study, the internal consistency reliability was Cronbach's α = .95; McDonald’s ω = .95.

#### 2.3.3 Defence styles

The Defence Style Questionnaire-40 [DSQ-40 (42);] evaluates defence style mechanisms through 40 items (e.g., ‘People say I tend to ignore unpleasant facts as if they did not exist’) on a Likert-type scale ranging from 1 (strongly disagree) to 9 (strongly agree). The DSQ-40 allows for assessing three main defence styles, including mature defences, (i.e., sublimation, humour, anticipation, and suppression), neurotic defences (i.e., undoing, pseudo-altruism, idealisation, and reaction formation), and immature defences (i.e., projection, passive aggression, acting out, isolation, devaluation, autistic fantasy, denial, displacement, dissociation, splitting, rationalisation, and somatisation). The DSQ-40 demonstrated satisfactory psychometric properties with sufficient construct validity and internal consistency reliability in the Italian context (42). In the present sample, the internal consistency reliability was Cronbach’s α = .62, McDonald’s ω = .62 for mature defences, Cronbach’s α = .57, McDonald’s ω = .56 for neurotic defences, and Cronbach’s α = .79, McDonald's ω = .79 for immature defences.

#### 2.3.4 Problematic social media use

The Bergen Social Media Addiction Scale [BSMAS (43);] estimates PSMU through 6 items (e.g., ‘How often during the last year have you felt an urge to use social media more and more?’) on a 5-point Likert-type response ranging from 1 (very rarely) to 5 (very often). The BSMAS showed excellent psychometric properties in terms of internal consistency reliability and construct validity (43). In this study, the internal consistency reliability was Cronbach's α = .81; McDonald’s ω = .81.

### 2.4 Statistical analysis

All statistical analyses were conducted using *IBM SPSS Statistics* for Windows, Version 26.0 (IBM Corp., Armonk, NY, USA). Descriptive statistics, including means and standard deviations, were computed for all study variables. Internal consistency reliability for all questionnaires was assessed using both Cronbach’s α and McDonald’s ω. The assumption of normality was assessed using the Shapiro–Wilk. To evaluate the potential impact of common method bias (CMB), Harman’s single-factor test was performed, adopting the critical threshold of ≥ 50% of the explained variance as an indicator of substantial bias (44, 45). Preliminary associations among variables were evaluated using partial bivariate correlations, controlling for sex. Mediation analysis was executed using Hayes’ (46) PROCESS Macro for SPSS version 3.5, applying Model 4, with 95% confidence intervals (CIs) based on 5,000 bootstrap samples. Bootstrapping is a non-parametric resampling procedure that constructs a large number of resamples of the same size as the original sample, drawn with replacement from the observed data, and recalculates the indirect effect in each resample to generate an empirical approximation of its sampling distribution (47). This method performs satisfactorily in a wide range of situations, including mediations, moderations, and moderated mediations (48, 49). An indirect effect is considered statistically significant when the 95% CIs do not include zero. Statistical significance was set at *p* < .05 for all analyses.

## 3 Results

### 3.1 Descriptive statistics and correlational analysis

The Shapiro–Wilk test indicated that all continuous variables (i.e., age, education, Borderline Personality, mature defences, neurotic defences, immature defences, and PSMU) were not normally distributed (Table 2).

**Table 2:** normality distribution (Shapiro–Wilk test) of continuous variables.

Variable	W
Age	.91***
Education	.32***
Borderline Personality	.93***
Mature defences	.99**
Neurotic defences	.99**
Immature defences	.99**
PSMU	.98***

*N* = 520. PSMU, Problematic Social Media Use, and W, Shapiro*–*Wilk Test. Statistically significant values in the Shapiro–Wilk test indicate that the variables are not normally distributed.

**p ≤ .01.

***p ≤ .001.

Descriptive statistics and partial correlations among the study variables are presented in Table 3. Results showed that age was not significantly correlated with any of the study variables. However, education was positively correlated with age (*r* = .35, *p* ≤ .001) and negatively correlated with immature defences (*r* = −.10, *p* ≤ .05). Borderline Personality was positively correlated with neurotic defences (*r* = .30, *p* ≤ .001) and immature defences (*r* = .55, *p* ≤ .001), while no significant correlations emerged with age, education, or mature defences. Finally, PSMU was negatively correlated with mature (*r* = −.21, *p* ≤ .001) and positively correlated with Borderline Personality (*r* = .47, *p* ≤ .001), neurotic defences (*r* = .17, *p* ≤ .001), and immature defences (*r* = .38, *p* ≤ .001).

**Table 3:** Descriptive statistics and partial correlations among study variables (controlling for sex).

Variable	*M*	*SD*	1.	2.	3.	4.	5.	6.	7.
1. Age	20.82	1.66	–						
2. Education	13.18	.97	.35***	–					
3. Borderline Personality	1.32	.88	.03	–.05	–				
4. Mature defences	5.47	1.20	.01	–.07	–.07	–			
5. Neurotic defences	4.81	1.25	–.05	–.03	.30***	.24***	–		
6. Immature defences	4.15	1.00	–.07	–.10*	.55***	.20***	.38***	–	
7. PSMU	2.52	.85	–.02	–.01	.47***	–.21***	.17***	.38***	–

*N* = 520. PSMU, Problematic Social Media Use.

*p ≤ .05.

*** *p* ≤ .001.

Harman's single-factor test showed that a single factor accounted for 27.11% of the total variance, below the conventional 50% threshold.

### 3.2 Mediation analysis

Mediation analysis was performed including Borderline Personality as the independent variable, PSMU as the dependent variable, and immature defences as the mediator. Age, sex, education, mature defences, and neurotic defences were included in the model as covariates. In the first stage of the model, Borderline Personality was positively associated with immature defences [*b* = 0.57, SE = 0.04, *p* < .001, 95% CI (0.49, 0.66)]. In the second stage of the model, immature defences were positively associated with PSMU [*b* = 0.21, SE = 0.04, p < .001, 95% CI (0.13, 0.29)]. The total effect of Borderline Personality on PSMU was significant [*b* = 0.42, SE = 0.04, *p* < .001, 95% CI (0.34, 0.49)]. Both the direct effect of Borderline Personality on PSMU [*b* = 0.29, SE = 0.04, p < .001, 95% CI (0.21, 0.38)] and the indirect effect through immature defences [*b* = 0.12, BootSE = 0.03, 95% CI (0.07, 0.18)] were significant. The full model explained 39% of the variance in PSMU, *R^2^* = .39. Table 4 summarises the mediation analysis.

**Table 4:** The mediation model linking borderline traits to PSMU via immature defences.

Path	*b*	SE	t	*p*	95% CI
Stage 1 (DV: Immature defences)					
Borderline → Immature defences	0.57	0.04	13.76	<.001	[0.49, 0.66]
Age	−0.04	0.02	−1.79	.07	[−0.08, 0.00]
Sex	0.08	0.07	1.19	.24	[−0.05, 0.22]
Education	−0.03	0.04	−0.77	.44	[−0.11, 0.05]
Mature defences	0.16	0.03	5.23	<.001	[0.10, 0.22]
Neurotic defences	0.15	0.03	4.83	<.001	[0.09, 0.21]
Stage 2 (DV: PSMU)					
Borderline → PSMU (direct effect)	0.29	0.04	6.63	<.001	[0.21, 0.38]
Immature defences → PSMU	0.21	0.04	5.30	<.001	[0.13, 0.29]
Age	−0.01	0.02	−0.56	.57	[−0.05, 0.03]
Sex	−0.18	0.06	−2.80	.01	[−0.30, −0.05]
Education	0.01	0.04	0.42	.68	[−0.05, 0.08]
Mature defences	−0.18	0.03	−6.35	<.001	[−0.23, −0.12]
Neurotic defences	0.03	0.03	1.01	.31	[−0.03, 0.08]
Total effect	0.42	0.04	10.70	<.001	[0.34, 0.49]
Indirect effect	0.12	0.03			[0.07, 0.18]

*N* = 520*.* DV, Dependent variable; PSMU, Problematic Social Media Use.

## 4 Discussion

Grounded in the I-PACE model (7–9) and the psychodynamic theory of defence mechanisms [Freud, 1894/1962 (26, 27);], the present study explored whether immature defences were involved in the association between Borderline Personality and PSMU among Italian young adults belonging to Generation Z.

Results supported the research hypothesis, showing that immature defences mediated the relationship between Borderline Personality and PSMU, after controlling for age, sex, education, mature defences, and neurotic defences. Overall, these findings suggest that immature defences may represent a key psychological process in the intersection between Borderline Personality and PSMU.

From a psychodynamic perspective, the present findings are consistent with the conceptualisation of immature defences as maladaptive strategies used to manage painful affect, interpersonal tensions, and inner conflicts [Freud, 1894/1962 (26, 27)]. Immature defences are characterised by distortion of inner and external reality, limited symbolic elaboration of affect, and a predominance of action-oriented responses to emotional distress (28). In individuals with high levels of Borderline Personality, who typically experience affective instability, heightened interpersonal sensitivity, fear of abandonment, and difficulties in maintaining integrated representations of self and others (24), these defence mechanisms may become particularly relevant. Within this framework, PSMU may be interpreted as a behavioural correlate of immature defensive functioning, in which compulsive and unregulated engagement with social media may serve as a rapid, externally oriented means of managing overwhelming emotional states (50). This interpretation aligns with previous evidence suggesting that individuals with high levels of Borderline Personality are more likely to engage in PSMU to obtain social approval, mitigate fears of rejection and abandonment, regulate intense affective fluctuations, and reinforce self-esteem and counter identity-related insecurity (18). Overall, these results support the view that PSMU may reflect a defensive solution to chronic affective instability rather than a simply reward-driven or compulsive and unregulated behaviour.

The present study contributes to the research based on the I-PACE model by integrating a psychodynamic layer into the understanding of PSMU. Within the I-PACE framework, immature defences can be conceptualised as regulatory processes operating at the intersection of the P, A, and C components. These defensive mechanisms reflect dynamic processes through which emotionally dysregulated states are managed, transformed, and ultimately expressed behaviourally. In particular, immature defences involve distortion of inner reality (e.g., unpleasant feelings) and external reality, limited symbolic elaboration of affect, and a predominance of action-oriented or perceptual strategies over reflective processing. In individuals with high levels of Borderline Personality, heightened emotional reactivity and interpersonal sensitivity may activate immature defensive responses that, in turn, shape affective and cognitive processing in response to social media stimuli.

The present findings also have potentially relevant clinical implications. Although the study was conducted in a non-clinical sample, the results suggest that prevention and intervention approaches for PSMU may benefit from considering both personality features and the defensive mechanisms underlying compulsive and dysregulated use of social media platforms. Specifically, interventions for individuals with specific personality features (such as Borderline Personality) and defence mechanisms could target the emotional and relational functions of social media use, helping individuals recognise when online engagement serves as an alternative for reflective processing, emotional containment, managing interpersonal uncertainty, and symbolising emotional distress. In this regard, PSMU may provide a context in which Borderline Personality and immature defences become more visible within therapeutic practice.

Despite these implications, the current research has some limitations that should be highlighted. The cross-sectional design precludes causal inference, and longitudinal studies are needed to clarify the temporal dynamics among Borderline Personality, defence mechanisms, and PSMU. In addition, the reliance on a non-probabilistic snowball sampling strategy, combined with online recruitment, introduces substantial selection bias and limits the generalisability of the findings. Moreover, all measures are self-report questionnaires, which may be subject to response biases, particularly when assessing largely unconscious processes, such as defence mechanisms. In addition, the internal consistency reliability for the mature and defence subscales was low in the present sample. Furthermore, relevant confounding variables, such as impulsivity and emotion regulation, have not been collected. Therefore, future studies should refine the variables of the model to comprehensively analyse the mechanisms underlying PSMU.

## 5 Conclusion

In conclusion, the present study advances the understanding of PSMU by highlighting the involvement of immature defence mechanisms in the association between Borderline Personality and PSMU. Specifically, the findings suggest that individuals high in Borderline Personality may be more prone to engage in PSMU as a consequence of maladaptive defensive processes involved in affect regulation. By integrating immature defence mechanisms within the I-PACE model, these findings underscore the importance of considering unconscious regulatory processes in PSMU, representing a promising direction for future research and intervention efforts.

## Data Availability

The raw data supporting the conclusions of this article will be made available by the authors, without undue reservation.
